# Supplementary figures and images for: iPHoP: An integrated machine learning framework to maximize host prediction for metagenome-derived viruses of archaea and bacteria
Source: PLoS Biol. 2023 Apr 21;21(4):e3002083. doi: 10.1371/journal.pbio.3002083 (PMC10155999; doi:10.1371/journal.pbio.3002083)

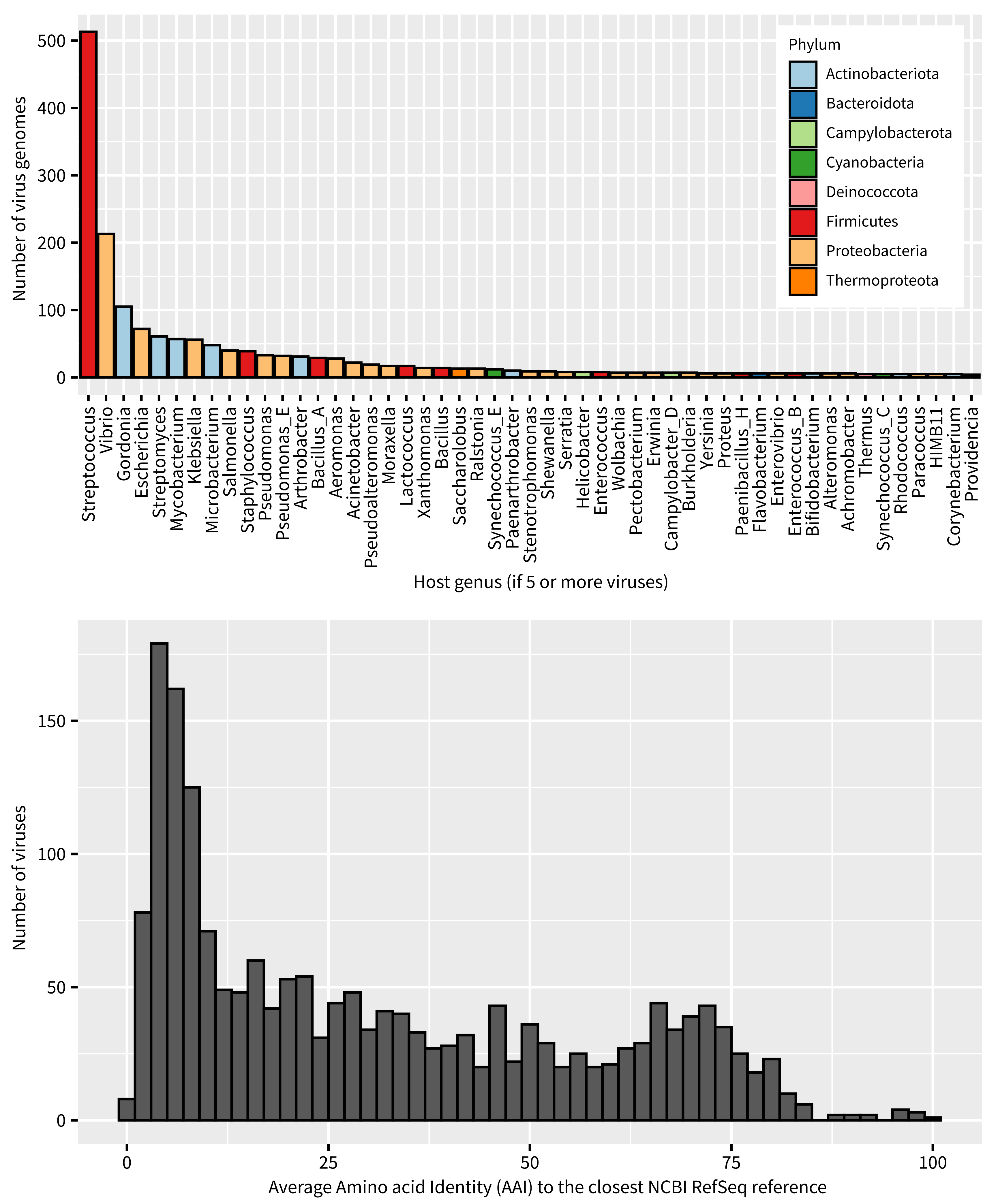

Supplement: S1 Fig — (A) Distribution of the host genera for the test dataset. Note: Only genera associated with ≥5 viruses are included, another 125 host genera were associated with <5 viruses and are not displayed. (B) Distribution of AAI to the closest reference in NCBI RefSeq for the test dataset. The corresponding list of viral genomes included in the test dataset is provided in S2 Table. Source data are available in S1 Data (Source data 1 and S2 Table). (TIF) [file pbio.3002083.s001.tif]

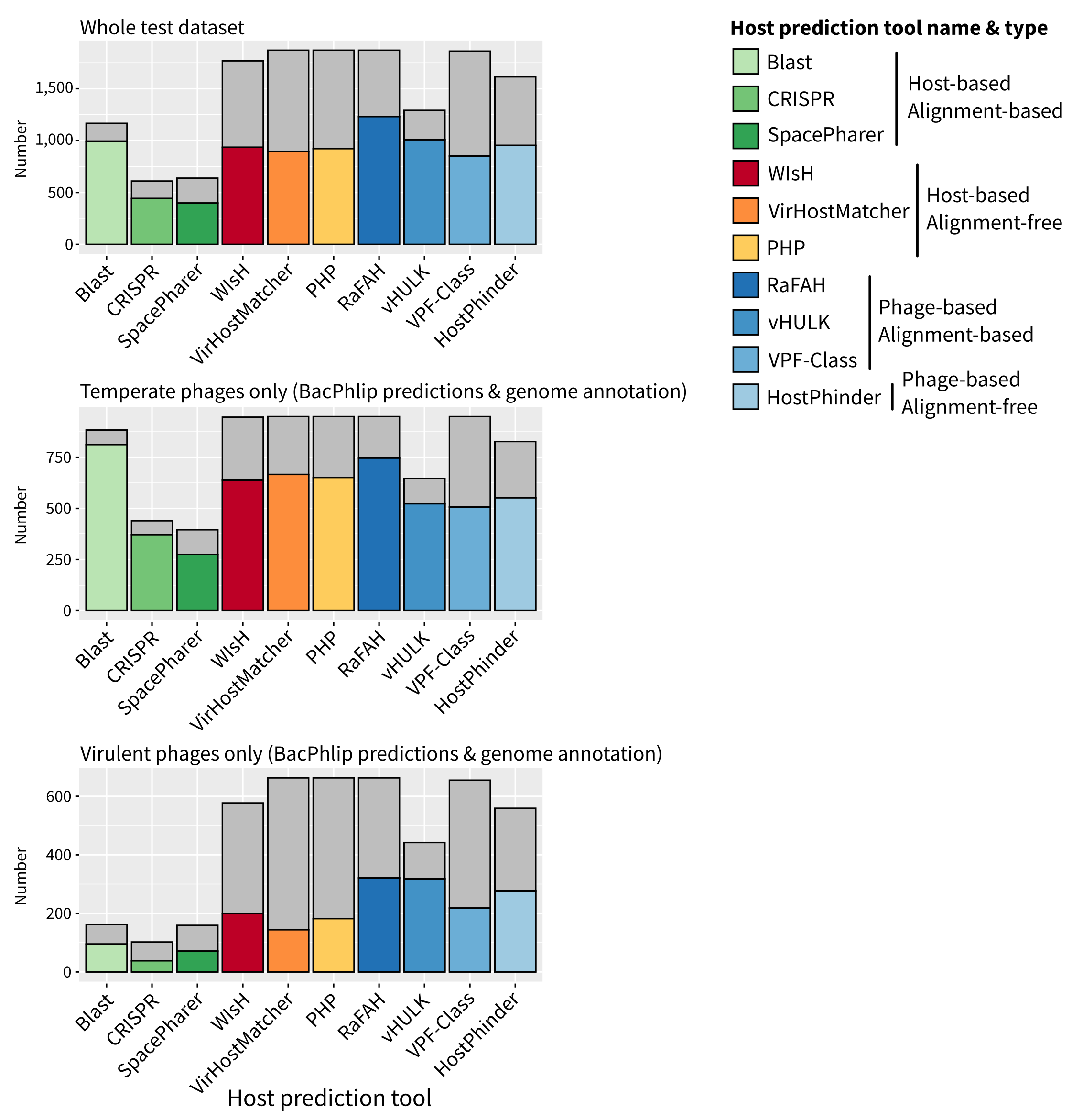

Supplement: S2 Fig — Total number of predictions and number of correct predictions (y-axis) obtained at any rank for each tool (x-axis) on sequences from the test dataset (S2 Table). For each tool, the number of correct predictions is indicated by the colored bar, while the total number of predictions is indicated by the gray bar. The top panel displays the results obtained on the entire test dataset (n = 1,870). The middle panel includes results obtained for all phages predicted as temperate, either via BacPhlip or based on the genome annotation (n = 949). The middle panel includes results obtained for all phages predicted as virulent by BacPhlip (n = 663). Source data are available in S1 Data (Source data 1). (TIF) [file pbio.3002083.s002.tif]

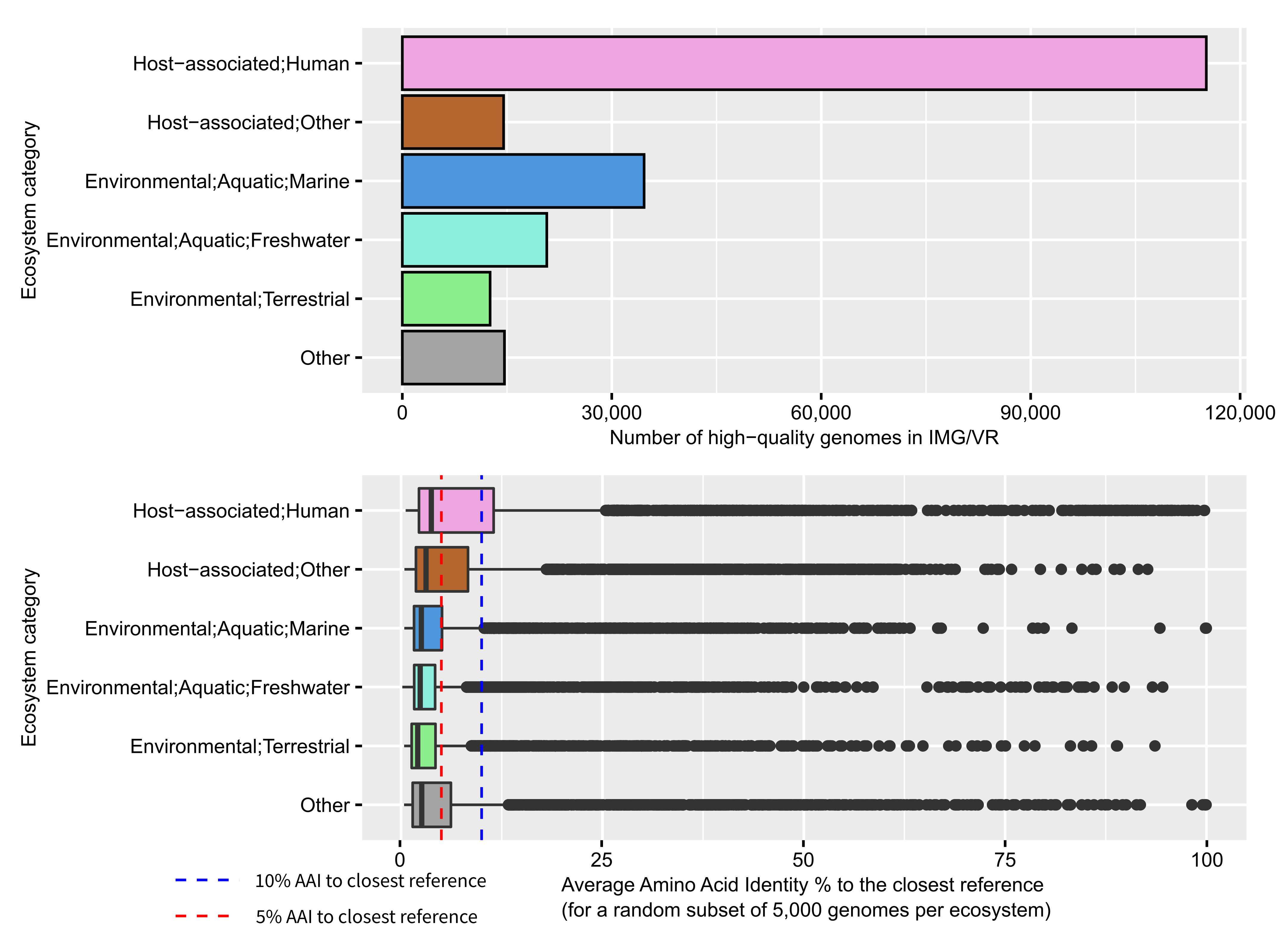

Supplement: S3 Fig — (A) Number of high-quality viral genomes from IMG/VR v3 identified across the 5 major biomes in the database. Genomes sampled from other biomes of lacking a biome information are gathered in the “Other” category. (B) Distribution of the average amino acid identity between IMG/VR v3 viral genomes and the NCBI Viral RefSeq v203. Source data are available in S1 Data (Source data 4). (TIF) [file pbio.3002083.s003.tif]

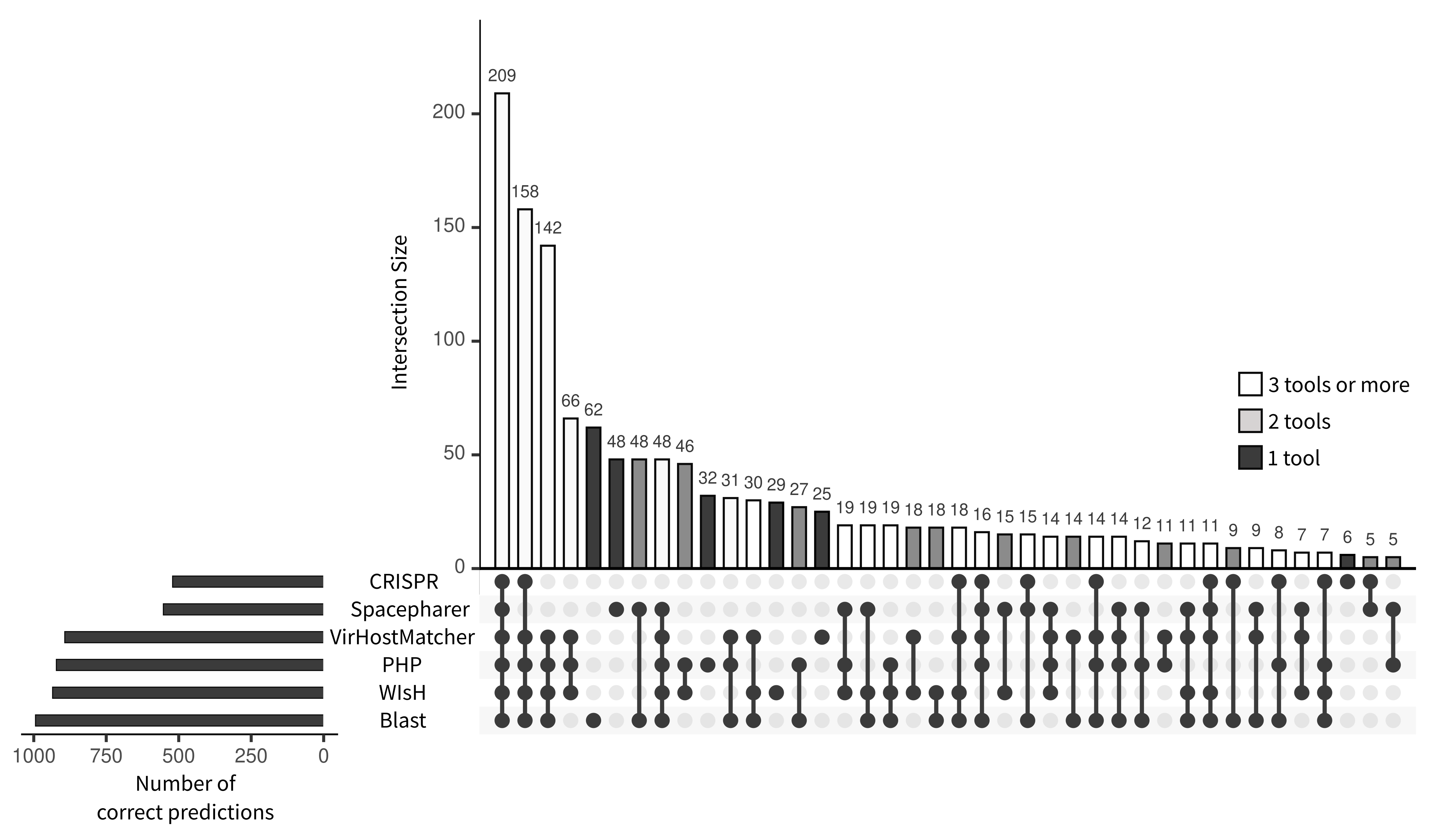

Supplement: S4 Fig — For each host-based tool included in the benchmark (see Fig 1), the overlap in terms of input sequence for which a correct prediction was obtained is presented here as an upset plot. The intersection size represents the number of phages with correct prediction using the combination of methods indicated at the bottom. This number is also indicated above each bar, and the bar color indicates the number of tools included in the combination. Source data are available in S1 Data (Source data 1). (TIF) [file pbio.3002083.s004.tif]

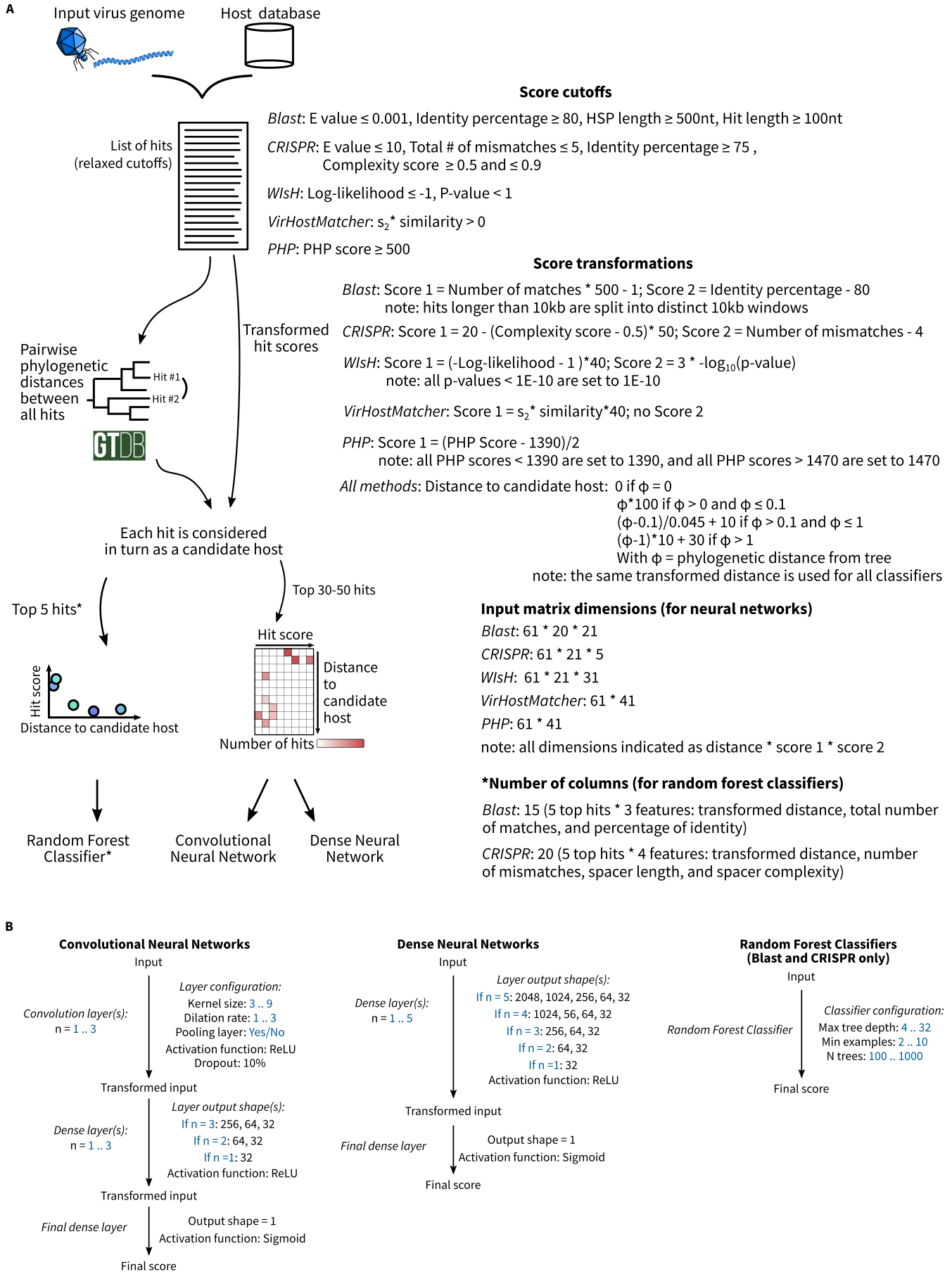

Supplement: S5 Fig — (A) Summary of the cutoff and metrics used for each host-based tool considered in iPHoP (see S1 Table). (B) Overview of the 3 different types of classifiers evaluated in iPHoP. The different parameters optimized using the Optuna framework are highlighted in blue. For varying numbers of layers, the same parameters were optimized for each layer, but each was optimized separately, i.e., the parameters values were independent between the different layers. (TIF) [file pbio.3002083.s005.tif]

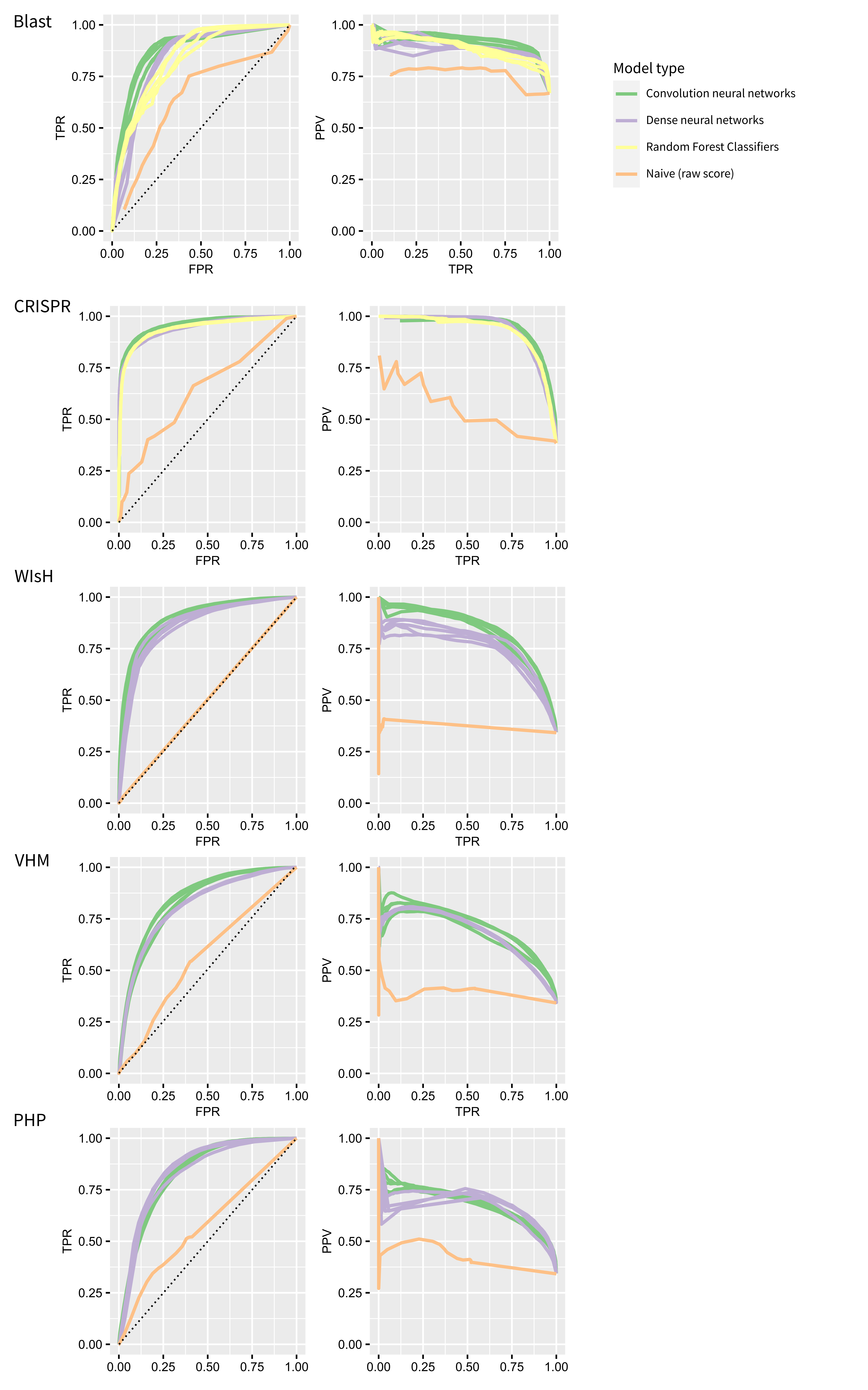

Supplement: S6 Fig — For each host-based tool, the ROC curves (left) and Precision-Recall curves (right) based on the test dataset are presented for the 5 best classifiers of each type and compared to the “naive” approach, i.e., best hit based on the raw score. FPR, false positive rate; PPV, positive predictive value; TPR, true positive rate. The 1-to-1 line is indicated as a dashed black line on the ROC curves. Random forest classifiers were only evaluated for Blast and CRISPR approaches. Source data are available in S1 Data (Source data 5). (TIF) [file pbio.3002083.s006.tif]

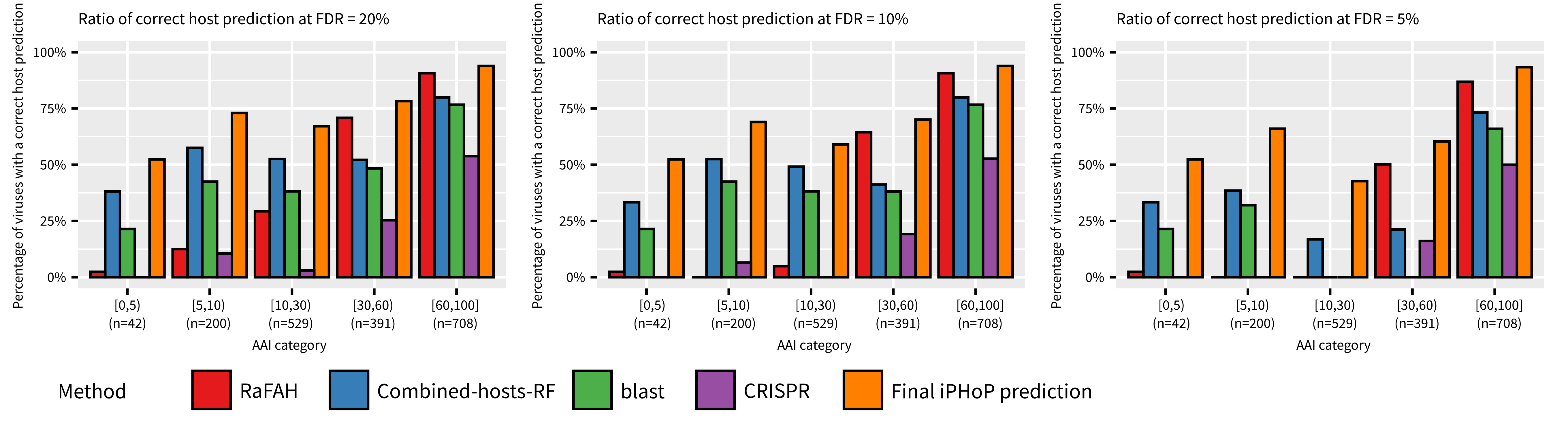

Supplement: S7 Fig — The number of correct host predictions was evaluated for 3 different score cutoffs corresponding to 20%, 10%, and 5% estimated FDR (false discovery rate). Input viruses were classified into 5 categories (x-axis) based on their AAI (average amino acid identity) to the closest reference phage genome. The number of correct host predictions is indicated for each iPHoP classifier (see Fig 3A) and for the composite score considering all classifiers (“Final iPHoP prediction”). Source data are available in S1 Data (Source data 3). (TIF) [file pbio.3002083.s007.tif]

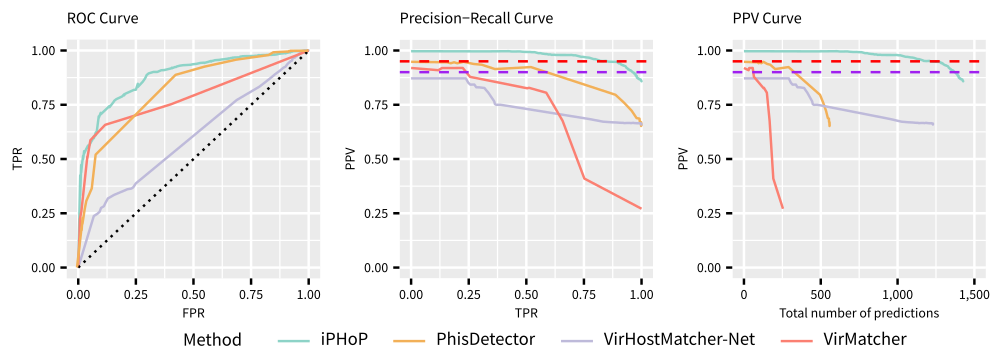

Supplement: S8 Fig — Standard Receiver Operating Characteristic (left) and Precision Recall (middle) curves for the 4 integrated host prediction approaches compared. To take into account the number of predictions provided by each tool, a third plot (right panel) indicates the positive predictive value (y-axis) when considering an increasing number of predictions (x-axis). To obtain this, cutoffs were progressively lowered to include an increasing number of predictions for each tool and prioritize the highest confidence ones, i.e., starting with the highest PPV possible. For the ROC curve, a 1-to-1 line is indicated with a dashed black line. For the Precision Recall and PPV curves (middle and right panels), the red and purple dashed lines indicate 5% and 10% false discovery rates, respectively. Source data are available in S1 Data (Source data 3). (TIF) [file pbio.3002083.s008.tif]

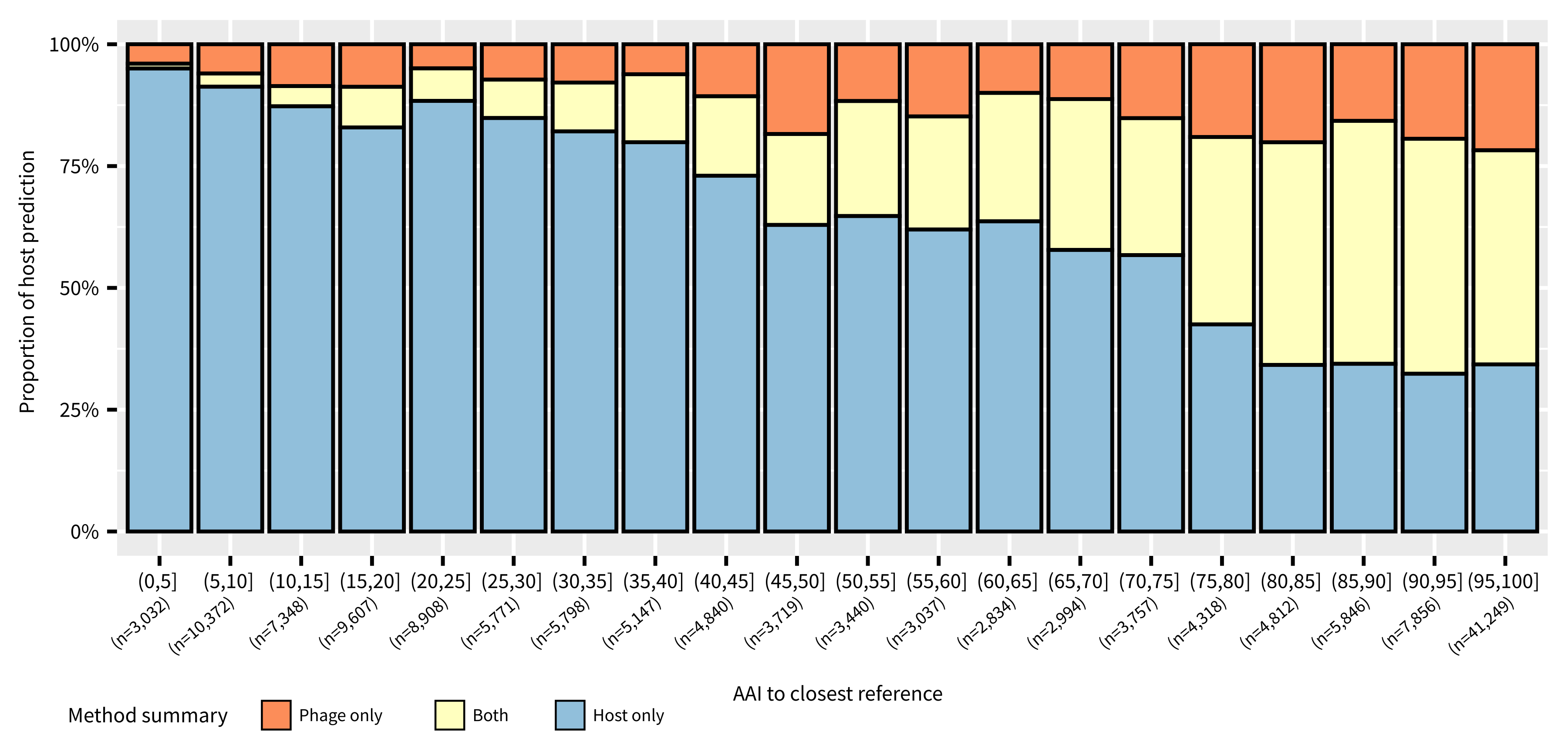

Supplement: S9 Fig — High-quality genomes from the IMG/VR v3 database for which a host prediction was obtained with iPHoP (score ≥90) were binned based on the average amino acid identity (AAI) to the closest reference in NCBI RefSeq Virus r203 (x-axis). Predictions entirely based on host-based tools are indicated as “Host only,” predictions exclusively based on RaFAH are indicated as “Phage only,” and predictions where both types of tools were consistent and with iPHoP score ≥90 are listed as “Both.” Source data are available in S1 Data (Source data 4). (TIF) [file pbio.3002083.s009.tif]

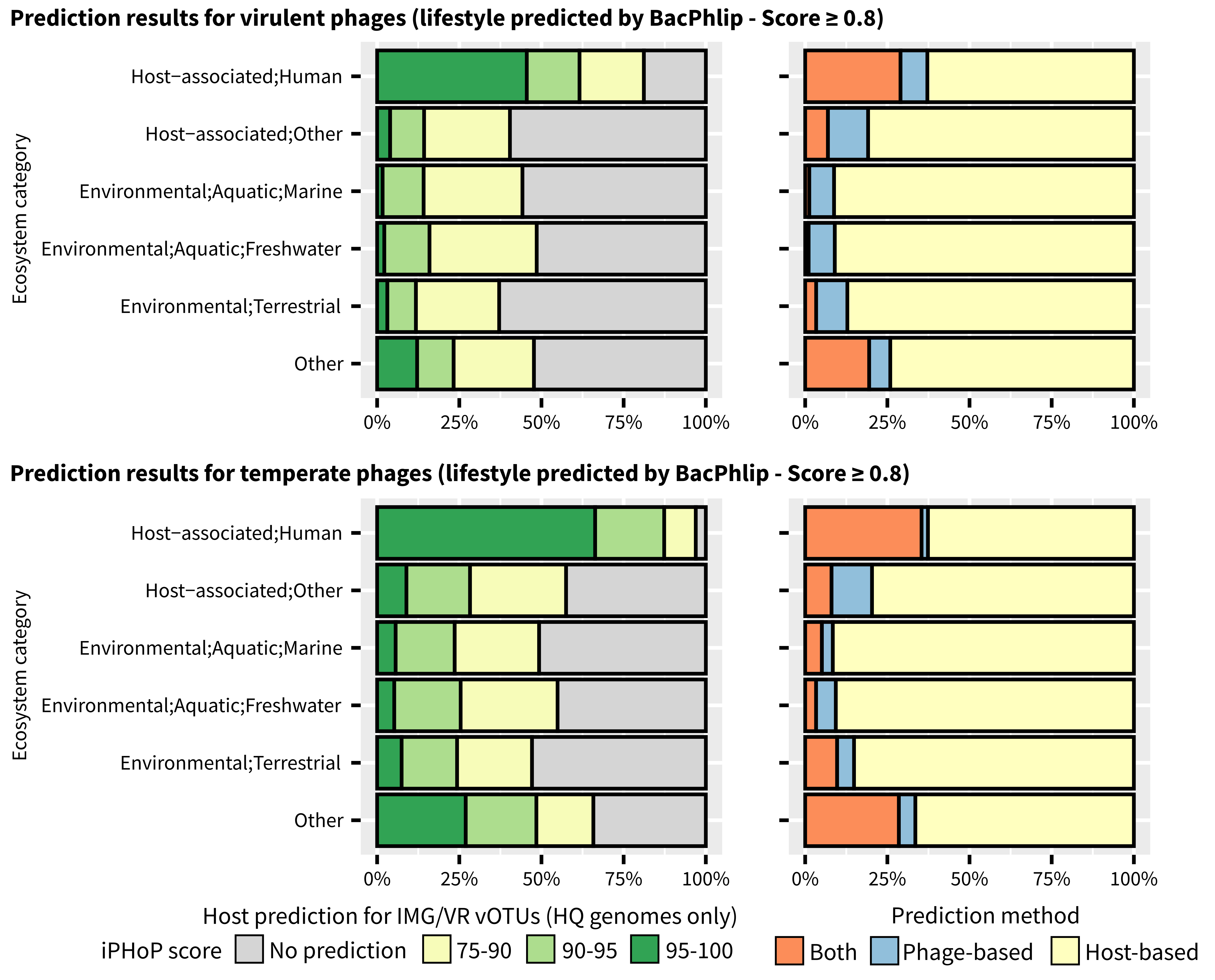

Supplement: S10 Fig — Breakdown of iPHoP host predictions for high-quality IMG/VR v3 genomes assigned as virulent (top) or temperate (bottom). Similar as Fig 4A and 4B, the left panel shows the distribution of the best score provided by iPHoP for the corresponding subset of IMG/VR v3 quality genome (top: virulent, bottom: temperate), organized by ecosystem. For each vOTU, the best score from iPHoP was considered if ≥75, or the vOTU was considered as not having a predicted host. The right panel shows the type of signal used to achieve host prediction with a score ≥90. “Host-based” includes all 5 host-based tools, while “Phage-based” includes predictions obtained with RaFAH. “Both” includes consistent predictions obtained with RaFAH and at least 1 host-based tool. Temperate and virulent phages were identified via BACPHLIP [55] with a minimum score of 0.8 and based on genome annotation (see Methods). Source data are available in S1 Data (Source data 4). (TIF) [file pbio.3002083.s010.tif]

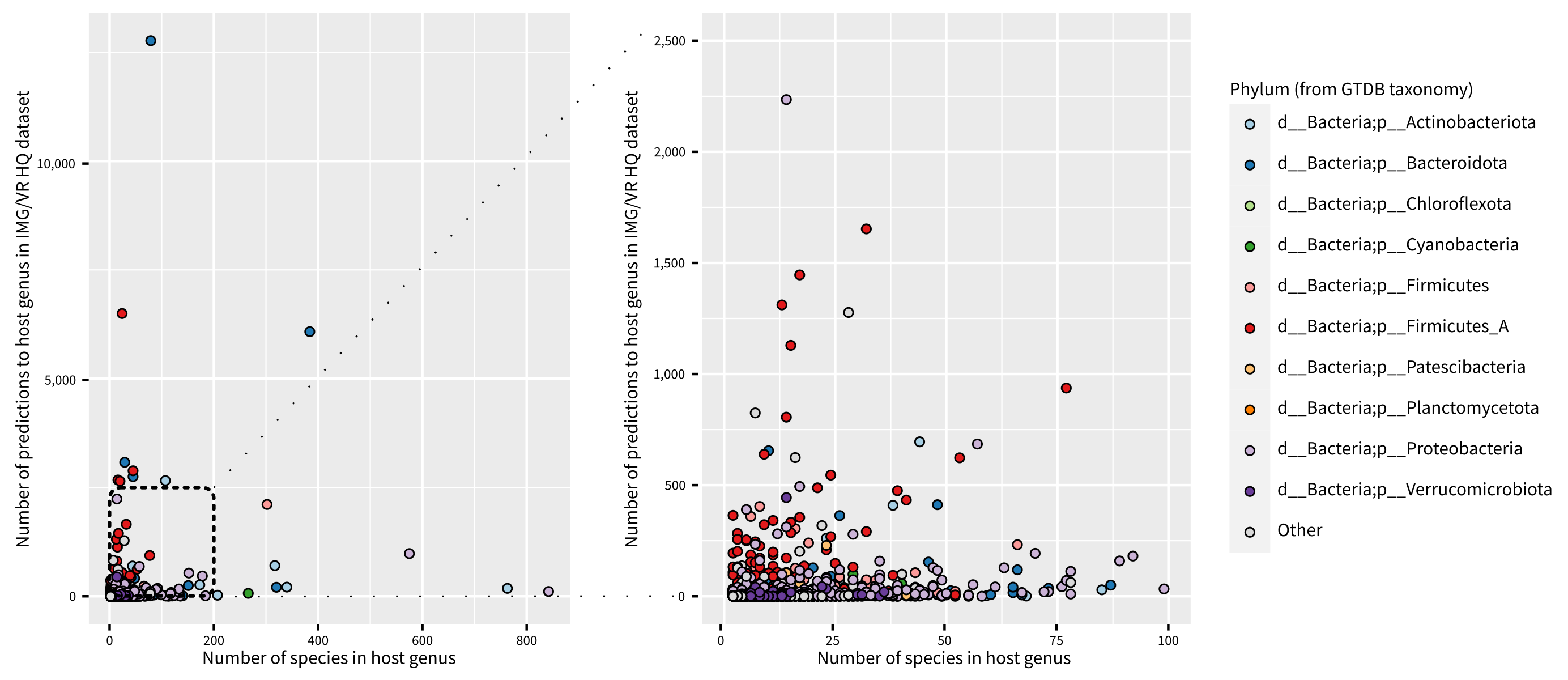

Supplement: S11 Fig — Each dot represents a host genus with at least 2 species, with the x-axis reflecting the total number of species in the genus, and the y-axis reflecting the total number of IMG/VR v3 HQ sequences predicted to infect this host genus with an iPHoP score ≥ 90. Host genera and species were obtained from the GTDB database [34]. The right panel presents a zoomed-in version of the area highlighted with dashed black lines in the left panel. Source data are available in S1 Data (Source data 4). (TIF) [file pbio.3002083.s011.tif]

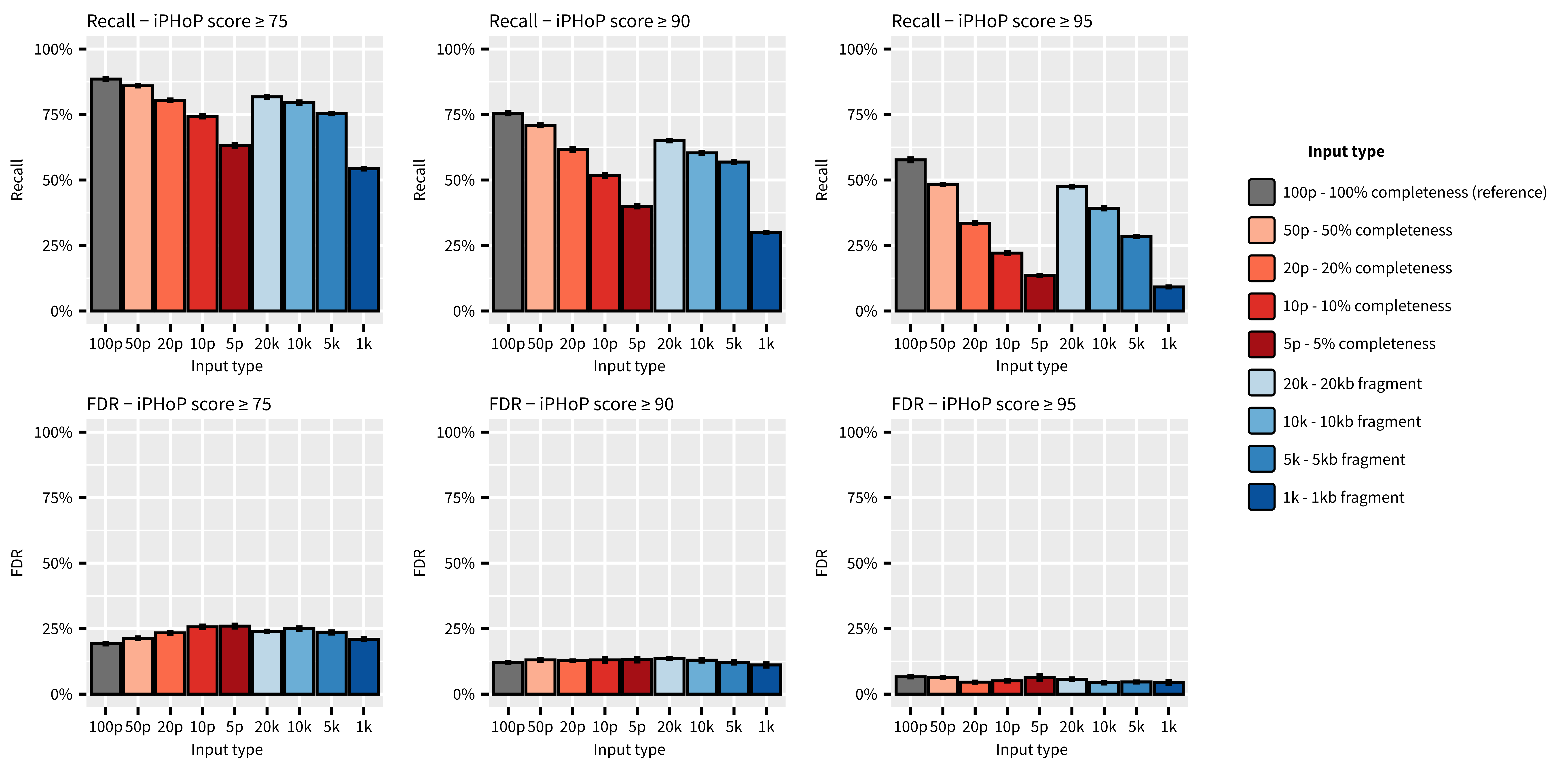

Supplement: S12 Fig — Partial genome assemblies were simulated by taking 500 random sequences from the testing set and selecting a random subset of fixed length (20 kb, 10 kb, 5 kb, and 1 kb) or fixed completeness (50%, 20%, 10%, and 5%), before processing with iPHoP for host genus prediction. This process was repeated 10 times, and the standard error across the 10 replicates is reported on each bar plot. Host prediction with iPHoP was also obtained for the same sets of 500 sequences from the testing set using complete genomes, for reference (“100% completeness,” colored in grey on the figure). For each input type, the recall (i.e., number of sequences with a host prediction, top), and FDR (i.e., percentage of erroneous predictions among all predictions, bottom) is indicated for different minimum iPHoP score cutoffs (75, 90, and 95). Source data are available in S1 Data (Source data 6). (TIF) [file pbio.3002083.s012.tif]

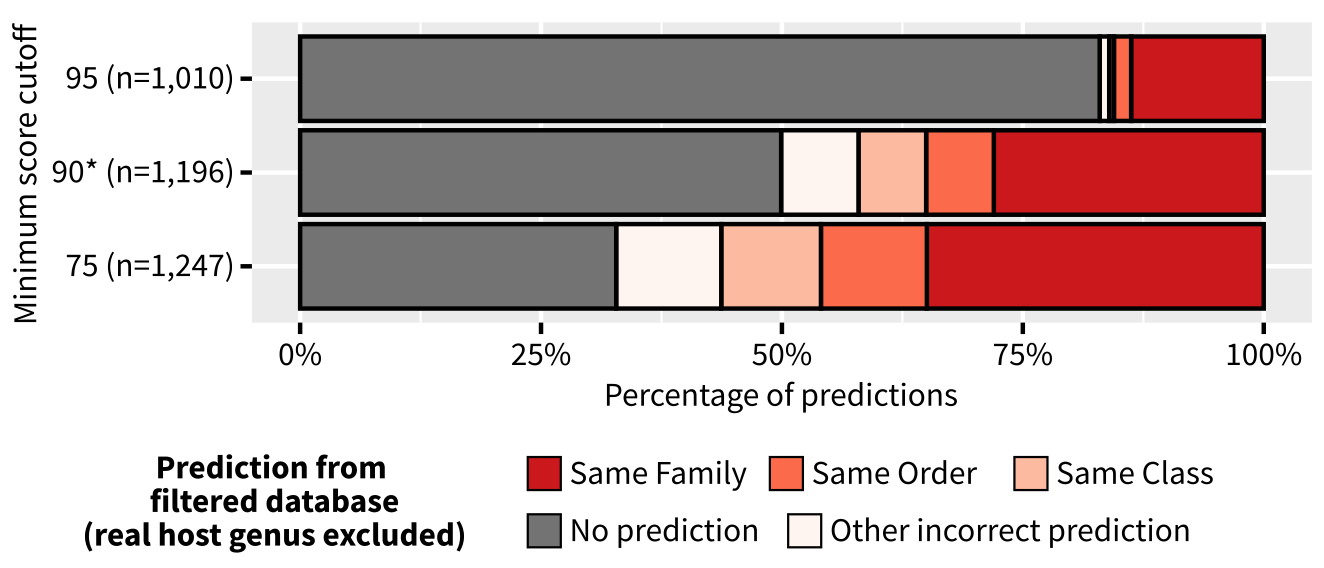

Supplement: S13 Fig — “Novel” host genera, i.e., cases in which the correct host was not represented in the host database, were simulated by recomputing iPHoP host prediction using filtered databases where all genomes from a given genus were removed. For this benchmark, only host-based predictions were considered and RaFAH phage-based predictions were ignored, as the latter did not rely on the availability of reference genomes in the host database. The same test dataset was used as in the regular benchmarks (see S1 Table and S1 Fig). For each minimum score cutoff (75, 90, and 95), all phages for which a correct host genus prediction was obtained with the standard database at this given cutoff were considered, and the prediction with the corresponding filtered host database was compared to the real host taxonomy. The category “no prediction” correspond to cases where iPHoP did not provide a prediction at the selected cutoff when using the filtered host database. The default score cutoff (90) is highlighted with a star symbol. The score cutoff of 75 is the lowest possible minimum score available in iPHoP. Source data are available in S1 Data (Source data 7). (TIF) [file pbio.3002083.s013.tif]
