# Supplementary material for: iPHoP: An integrated machine learning framework to maximize host prediction for metagenome-derived viruses of archaea and bacteria
Source: PLoS Biol. 2023 Apr 21;21(4):e3002083. doi: 10.1371/journal.pbio.3002083 (PMC10155999; doi:10.1371/journal.pbio.3002083)
Supplement: Attachment — Submitted filename: iPHoP_R2R_R1_1.1.pdf [file pbio.3002083.s022.pdf]

Note: Line numbers refer to the “highlighted_changes” pdf version of the revised manuscript.

Reviewer #1: This is an integrated, multistep approach to adjudicate viral genomes from metagenome datasets to their putative host. The work is well executed, and the draft is carefully written. Below, there are some comments that aim at pressure testing some of the central statements.

1.1 Title: The title refers to "metagenome assembled virus genomes" - In reality it should refer to phage viral genomes.
Response: Unfortunately, neither “virus” or “phage” are perfect here, because “phage” only refers to bacteriophages while iPHoP also applies to archaeoviruses, but on the other hand “virus” also includes eukaryotic viruses which may be misleading since iPHoP does not apply to these. For clarity, we changed the title to “metagenome-derived viruses of archaea and bacteria”, and specified in the new overview schematic (Fig. 1D) that the input for iPHoP consists of “metagenome-derived bacteriophages and/or archaeoviruses”. We also clarified “a broad range of viruses” as “a broad range of viruses infecting bacteria and archaea” in the abstract (l. 25).

1.2 Abstract: The manuscript carefully refers to host genus predictions. The abstract should be more precise - currently it refers to "host predictions" indicating "reliable host predictions at the genus rank" would be adequate.
Response: We thank the reviewer, and have adjusted the abstract l. 24: “to reliably predict host taxonomy at the genus rank”

1.3 On lines below 246: There is an important discussion on the presence of cognate host sequence in the material from where the metagenome was sequenced ("local"). Certainly, co-occurrence of the phage and the putative genome is a valuable input. Overall, a substantial (>25%) proportion of MAGs used for host predictions were obtained from metagenomes generated in the same study from which the input phage was derived. It is unclear how this information was used to evaluate performance of iPHoP. There is an analysis described below line 269, but it is unclear that it reaches the value of developing models that are "local" aware. The authors are fully aware of the potential ("increasing the diversity of isolated phage-host pairs from various environments will likely help refine these predictions in the future") but is not clear that they have used available data to that goal.
Response: We thank the reviewer for the suggestion. In response to a similar comment from reviewer #2, we now provide additional benchmarks, including a comparison of iPHoP prediction with and without these MAGs (see detailed response to question 2.4 below). In addition, we also clarified that the default iPHoP database includes 59,546 MAGs. primarily from the GEM dataset (doi 10.1038/s41587-020-0718-6), which helps to generate more host predictions especially in marine and freshwater samples: “Given this increased prediction rate, public MAGs from uncultivated bacteria and archaea (~ 60,000) were included in the iPHoP default host database” (l. 256).

1.4 One of the critical aspects of machine learning approaches is the management of "leaking" from training sets to test sets. Here, there are a number of approaches aiming at controlling for such bias. However, it is unclear how for example IMG/VR phage sequences and pairs were checked for absence of the datasets used during training.

Response: We agree with the reviewer that controlling the "leaking" between training and test sets is critical. To illustrate that the IMG/VR phage sequences were not present in the datasets used in training, we now report a comparison between the two datasets using genome-wide AAI. Specifically, 90% of the IMG/VR datasets shared < 10% AAI with their closest relative in the training and test sets. This number was 87% when considering only IMG/VR sequences with an iPHoP prediction suggesting that similarity to the reference was not a substantial bias in this analysis. This is now clarified in the text on l. 643: "To verify that this dataset did not overlap significantly with the training and test sets, genome-wide AAI was computed between the 216,015 high-quality genomes and the genomes included in the training and test sets using the AAI estimation script provided with the Metagenomic Gut Virus catalogue (https://github.com/snayfach/MGV/blob/master/aai_cluster/). Overall, 90% of all IMG/VR v3 sequences, and 87% of IMG/VR v3 sequences with at least 1 host predicted, displayed less than 10% AAI to their closest reference in the training or test sets, confirming that the IMG/VR sequences are meaningfully distinct from these training and test sets."

1.5 Figure 1 shows the comparison of different host prediction approaches on a single test dataset. It is placed early in the text, before iPHoP is presented. The issue with this mode of drafting the manuscript is that there is no formal comparative of the new tool here. It is true that there are a number of comparative plots later in the manuscript but are more selective. More generally, it is difficult to assess in a first read how much of the tool goes beyond integrating existing tools, using ensemble of machine learning approaches (eg., Figure 2), or using composite scores (eg., Figure 3), and when and how the taxonomy aware improvements are included. The work would benefit from an end-to-end view of the process of iPHoP. And as indicated above, clarity on what is used as truth.
Response: We thank the reviewer for the suggestion. We do not believe that comparing iPHoP with individual approaches listed in Figure 1 would be fair, since iPHoP relies on several of these different approaches considered together. However, we understand that Figure 1 only presenting individual tools and not iPHoP itself makes it difficult to grasp where this new tool (iPHoP) fits, and what are its different components. To help clarify this aspect, we added a new panel D to Fig. 1 that provides a schematic end-to-end view of iPHoP, and highlights specifically which steps are newly developed for iPHoP as opposed to relying on existing tools.

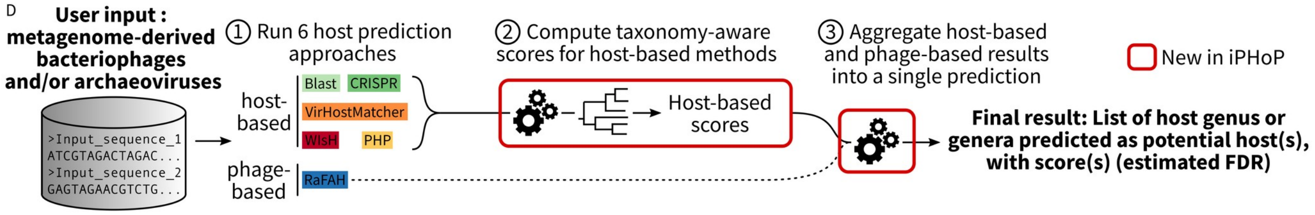

<u>Reviewer #2</u>: The authors present iPHoP, a computational framework to associate metagenome-derived viral genomes with microbial hosts. Existing methods leverage both alignment-based and alignment-free tools to predict phage hosts, though these approaches suffer from limited taxonomic resolution and / or high false-discovery rates. iPHoP refines the output of such tools to yield genus-level associations that are both sensitive and specific, giving insight into the putative hosts of phage, including "novel" phage with low sequence similarity to phage with known hosts. When applied to metagenome-derived phage genomes the IMG/VR database, iPHoP increases the proportion of phage with host assignments by

more than 10-fold for certain ecosystems. The authors also create a database building module, Bioconda recipe and a Docker container for users to apply iPHoP to their own datasets.

Major Comments

2.1 Since the main usage of this software will be on metagenomics datasets, it would be great to have the authors benchmark and spend more time on those types of datasets. The authors use MAGs from a sample to predict the virus-host associations of viruses within that same sample (section starting 255). Which tool was used in the first step of IPHoP?

Response: We thank the reviewer for the comment and the suggested additions, and agree that the manuscript would benefit from additional description. As an answer to a comment from reviewer #1, we have now added a panel D to Fig. 1 that clarifies the overall pipeline used by iPHoP, including which specific tools are used in the first step.

2.2 Since so many of the associations (~30%) were from the MAGs alone, it seemed like there should be more benchmarking of this pipeline. For one, it relies on using BACPHLIP as a phage-finding method. Why was this picked and how accurate precise is it?

Response: We agree with the reviewer that the manuscript would benefit from additional analysis of the metagenome-assembled genomes. First, we would like to clarify that all the metagenome-derived sequences used in this manuscript were derived from the IMG/VR v3 database, and thus were already identified as viral using the Viral Sequence Detection Pipeline as previously published (doi: 10.1093/nar/gkaa946). In our study, BACPHLIP was only used to predict whether these phages were likely lytic or temperate. This has now been clarified l. 636 as follows: "These sequences were previously identified as prokaryotic virus genomes, i.e. all viruses predicted to infect eukaryotes were excluded, by the IMG/VR v3 pipelines.".

2.3 Importantly, how do the other host-annotation prediction algorithms do on this dataset? How much do their predictions overlap?

To address this question, we applied VirHostMatcher-Net to the same IMG/VR dataset and report on how the results differ from iPHoP predictions. Unfortunately, we could not do the same for PhisDetector or VirMatcher because these tools did not scale to the processing of ~ 200,000 sequences. Overall, using cutoffs leading to a similar False-Discovery Rate (~ 15%), we observed that VirHostMatcher-Net yielded a host prediction for 49,870 sequences, compared to 130,867 predictions for iPHoP, In addition, of the 49,870 sequences with a VirHostMatcher-Net prediction, 43,124 also had a prediction via iPHoP. We now report this additional test in the manuscript as follows l. 239: "For comparison, VirHostMatcher-Net applied to the same dataset yielded a host prediction for 23% of the input sequences for a comparable estimated FDR of 15%, with the majority (86%) of these sequences also being connected to a predicted host via iPHoP.".

2.4 What would have been the results without the input of the additional MAGs?

To address this question, we performed two additional benchmarks on the IMG/VR sequences.

First, we built a custom host database which did not include any MAG, and used it in a iPHoP host prediction of the same IMG/VR sequences. This indicated that ~ 10% of IMG/VR sequences with a host prediction from the full host database did not yield a host prediction with the "no MAG" database. This "prediction loss" was highest for marine (17%) and freshwater (20%) sequences, consistent with the higher proportion of host MAG genomes

used in these predictions. This is now reported in the text as follows: "This was confirmed by performing host prediction with iPHoP for the same sequences using a custom database without any MAG, which yielded 9.95% less host prediction on average, including 17.65% and 20.71% less for freshwater and marine phages, respectively (Table S5). Given this increased prediction rate, public MAGs from uncultivated bacteria and archaea (~ 60,000) were included in the iPHoP default host database." (l. 256).

Then, for 3 freshwater studies, we built custom databases in which the "local" MAGs, i.e. MAGs from the same study, were removed. This indicated that ~ 26% of viruses had a host prediction with the full host database but not with the host database lacking the "local" MAGs. We now report this additional benchmark in the text as follows: "We confirmed the relevance of "local" MAGs by performing host prediction with iPHoP for 3 specific studies using custom databases where host MAGs from these studies were removed, which resulted in 26% of the host predictions being lost." (l. 264).

Finally, we performed and report results for two additional benchmarks in a new section: "Partial genomes and eukaryotic viruses as potential sources of errors".
First, we analyzed how contig length and/or completeness impacted iPHoP performances, as a key feature of metagenome datasets is that they typically are a mix of complete and partial genomes. Specifically, we randomly selected subsets of sequences from the testing set at specific length (20kb, 10kb, 5kb, 1kb) or completeness (50%, 20%, 10%, 5%), and used iPHoP to predict host taxonomy for these partial sequences. Overall, the recall (i.e. number of sequences with a prediction) decreased as the sequence length/completeness decreased, especially when reaching ~ 5% completeness or 1kb. However, the False-Discovery Rate stayed stable at "high" cutoffs (minimum score 90 and 95) and only slightly increased at minimum score 75. Said otherwise, partial genomes lead to a lower rate of hosts predicted by iPHoP, but not a significant increase in erroneous predictions. This is now indicated in the main text as follows: l.310 "When applying iPHoP to randomly fragmented sequences from the test set, we observed a systematic decrease in recall with decreased length and/or completeness (Supplementary Fig. S12). However, the FDR observed for these partial genomes was similar to that observed for complete genomes for "high" cutoffs (minimum iPHoP score 90 and 95), and only slightly increased at minimum iPHoP score 75 (from ~20% to ~25%). Taken together, this suggests that applying iPHoP to partial genomes is likely to result in reduced recall, i.e. fewer input sequences will receive a prediction with a high score, but the number of erroneous predictions is unlikely to increase substantially."

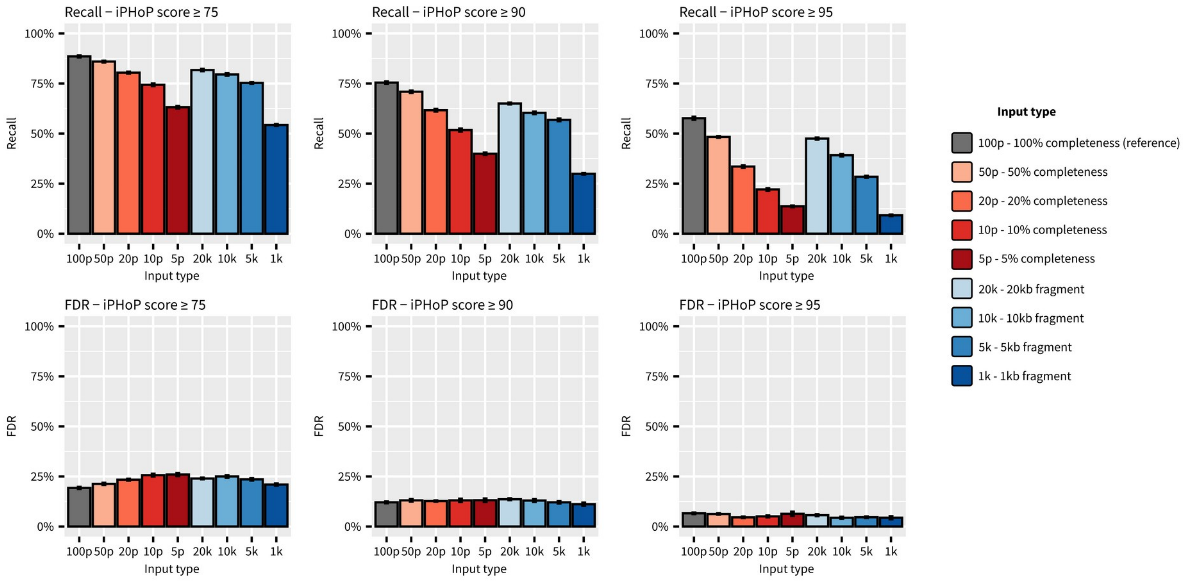

Similarly, we explored how iPHoP would handle viruses known to infect eukaryotes. To that end, we applied iPHoP to 8,128 eukaryotic virus genomes obtained from RefSeq r214. Overall, iPHoP (wrongly) predicted a bacterial or archaeal host for 1,018 of these viruses, most (90%) with a low score (< 90). These predictions were mostly based on k-mer similarity to bacteria and archaea genomes, and mostly obtained for viruses with short genomes in the Riboviria (RNA viruses) and *Monodnaviria* (ssDNA viruses) phyla. To help guide users who may attempt to process this type of virus with iPHoP, we now report these results and recommend adding a "host prediction domain" step prior to iPHoP, which can be based either on a taxonomic assignment of the virus or on automated tools like Host Taxon Predictor: "Applied to 8,128 eukaryotic virus genomes from RefSeq, iPHoP predicted a bacterial or archaeal host for 1,018 viruses (Table S7). These erroneous predictions were primarily (85%) derived from the k-mer comparison to bacteria and archaea genomes, for relatively short viral genomes within the RNA viruses (Riboviria realm, 640 – 63%) and ssDNA viruses (Monodnaviria realm, 155 – 15%), and the vast majority (90%) were associated with a relatively low confidence score (iPHoP score < 90). These results warn that false-positive predictions may occur when users apply iPHoP to a mixed dataset containing both prokaryotic and eukaryotic viruses, especially for samples composed of RNA and/or ssDNA virus sequences. To alleviate this potential issue, we recommend users to cross-reference any iPHoP prediction with a host domain prediction, i.e. a prediction of whether the sequence belongs to a prokaryotic or eukaryotic virus, based on taxonomic assignment of this virus or on an automated tool such as Host Taxon Predictor(46)." (l. 317).

2.5 The authors mention RaFah as having good overall performance. Since iPHoP runs as a refinement tool, why did the authors not apply it to RaFah as well? The authors show that it can improve the predictions of host-based methods (Figure 3).
Response: The reviewer is correct, RaFAH already provides reliable predictions, and was not refined as the host-based tools were. This was largely because most of the improvement provided by this "refinement" step is already included in RaFAH: while host-based tools yield hit(s) to individual genomes, RaFAH was originally designed to predict host taxonomy at the genus rank, and therefore can not (and does not need to) be made "taxonomy-aware" via the same process as was done for the host-based methods. This has been clarified in the new schematic (Fig. 1D).

2.6 Figure S7 nicely shows an improvement over RaFah, but what host prediction tool was used in the primary step here? Since the percentage of prophage correctly assigned is highest for the combined and in almost all cases, results in a more than 10% increase, perhaps the prescription is to run all of these methods or at least some combo of them? Additionally, could the outputs of multiple tools be used as input for iPHoP?
Response: The reviewer is correct, and we apologize for the lack of clarity. "Combined" here meant using all the other methods aggregated using the new iPHoP classifier (step 3 in the new schematic, Fig. 1D), and is the default process of iPHoP. This has now been clarified in the new Fig. 1D.

2.7 Concerning Figure 1, "correct" host predictions are assessed at the genus rank (line 105). It would be informative to have more taxonomic categories for Figure 1A than "correct" and "incorrect" predictions. For example, predictions may be "correct" at the ranks of family or class, and this information would be beneficial to understanding which existing tools are "close to correct" and which are way off the mark, even if the rest of the manuscript only considers predictions at the genus level.

Response: We thank the reviewer for the helpful suggestion. We have now added a new supplementary table summarizing the benchmark results for individual approaches (Fig. 1) at different ranks and for different score (Supplementary Table S3). As could be expected, the percentage of correct predictions was higher for higher ranks (e.g. phylum and class), compared to genus. However, as the reviewer suggested, there were clear differences between tools at the different tanks, which is a relevant information to share with the reader.

2.8 iPHoP seems to be able to predict multiple hosts, if they exist (for example, what Fig 2B shows). Can the authors expand the performance of various callers and their own as a function of number of hosts?
Response: We agree with the reviewer that, on principle, iPHoP (and potentially other callers) could identify cases of phages with multiple hosts across different genera. Unfortunately, however, we are not aware of any dataset of such broad host range phages that would be robust and large enough to be used here for benchmarking. Since this question represents one potential avenue for improvement, we now mention this in the discussion l. 357: "several potential improvements to iPHoP can already be envisioned, including for instance the addition of complementary approaches such as the detection of shared tRNA between phages and hosts, the consideration of additional features such as whether the input virus is temperate or virulent, and the ability to predict multiple hosts for broad host range phages."

Minor comments:
2.9 Figure 5A is difficult to interpret given the different phyla shapes. Could the authors include numbers or a log-scale bar graph and/or color scale with the different categories for phage association?
Response: We thank the reviewer for the helpful suggestion. We have now added a new supplementary table with the underlying data for Fig. 5A (Supplementary Table S6).

2.10 Why the distinction for genera with at least 2 host species? (line 258)
Response: We considered that genera composed of a single species were mostly novel, not well represented in the current database, and thus potentially skewing the results when looking at the overall percentage of genera associated with a virus. We agree with the reviewer however that these data for genera with only 1 host species are potentially interesting, and we now included these in a separate tab in the new supplementary table (Supplementary Table S6).

2.11 Supplementary Table 1 is useful. In Fig 1C, there is apparent bias for closely related phages when predicting hosts with alignment-free phage-based tools (HostPhinder). Why is assigning hosts by phage-phage k-mer similarity apparently dependent on AAI%, whereas assigning hosts by host-phage k-mer similarity is more-or-less agnostic to the level of phage novelty?
Response: This is a good question, and we would argue there is not a clear answer yet. Based on these benchmarks (and in agreement with the findings in the original HostPhinder paper), it seems like the phage-phage k-mer similarity reflects primary sequence similarity between query and references, and as such will be directly correlated with similarity to the reference sequence at the nucleotide and amino acid level (AAI%). Meanwhile, the host-phage k-mer similarity seems to reflect more an overall nucleotide composition similarity, most likely stemming from adaptation in terms of e.g. GC content and codon usage, and would not be correlated to amino acid similarity to references in that case. While we can not definitively answer the question, we have now clarified in the text that the reference bias

seems to impact even tools relying on phage-phage k-mer similarity: “This reference bias was observed for all phage-based tools, including the one based on k-mer similarity (HostPhinder, Fig. 1C)” (l. 133)
