# Supplementary material for: iPHoP: An integrated machine learning framework to maximize host prediction for metagenome-derived viruses of archaea and bacteria
Source: PLoS Biol. 2023 Apr 21;21(4):e3002083. doi: 10.1371/journal.pbio.3002083 (PMC10155999; doi:10.1371/journal.pbio.3002083)
Supplement: Attachment — Submitted filename: iPHoP_R2R_R2.pdf [file pbio.3002083.s023.pdf]

Note: Line numbers refer to the “highlighted_changes” pdf version of the revised manuscript.

The remaining point refers to false positives, you should discuss your approach to reduce false positives taking into account the fact that there is no guarantee that the host genome is present in the host-db. E.g., what would happen if all members of the host genus are removed from the input? Would the method predict some random genus, a closely related genus, or nothing? We think that would be a more realistic evaluation of false positives in the metagenomic context.

Response: Apologies again for missing this request in the first round of revision. We have now performed these benchmarks on the same test set by selectively removing individual host genera from the database, running iPHoP with these custom database, and evaluating the host prediction obtained for the different phages. Overall, iPHoP either did not provide a host prediction or provided a prediction to another genus in the same family. However, at lower scores (< 90), a larger number of phages were associated with predict hosts more distantly related to their original host, sometimes in a different class or phylum. We thus cautioned future users about the interpretation of such low scores when processing datasets where host genera are expected to be missing from the reference genome database. These data are presented in a new Fig. S13:

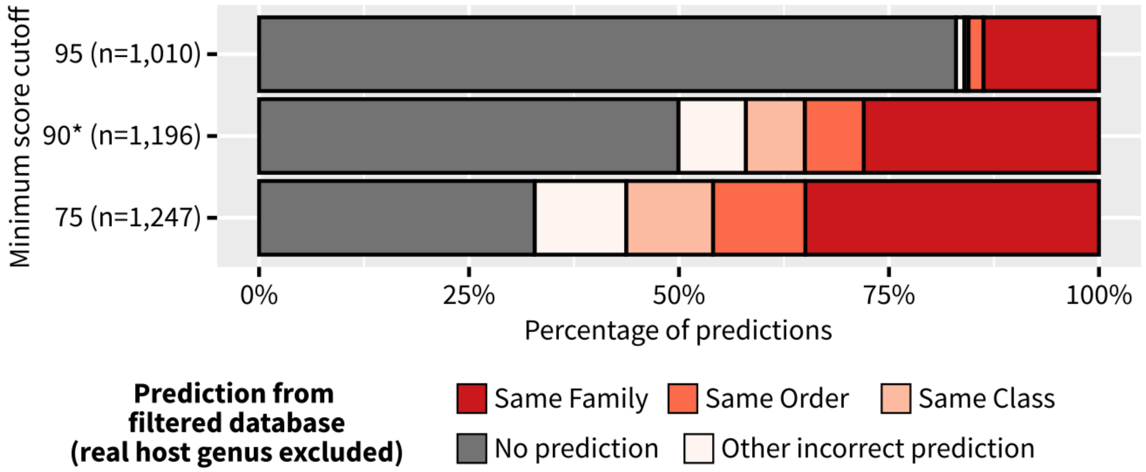

and presented in the main text as follows (l. 313): “Next, we evaluated the results of iPHoP host prediction for phages infecting “novel” hosts, i.e. a host genus without a representative genome in the host database. To that end, we applied iPHoP on the test set using filtered databases in which all reference genomes from the real host genus were removed (Supplementary Figure S13). Overall, when using minimum score cutoffs of 90 or 95, the majority of phages did not yield a prediction when the real host genus was filtered from the database, and when a prediction was provided, it was to a genus in the same family as the real host genus. For a more relaxed minimum score cutoff of 75, iPHoP provided a prediction for 67% of the input phages, including to genera in the same order or class as the real host (Supplementary Figure S13). This suggests that, when processing samples with a large number of novel host genera, i.e. not represented in the current reference genome databases, iPHoP predictions should ideally be restricted to “high” score (i.e. score ≥ 90) and interpreted at the family rank.”

Please ensure that you provide the individual numerical values that underlie the summary data displayed in the following figure panels as they are essential for readers to assess your analysis and to reproduce it: Figures 1ABC, 2CD, 3BC, 4, 5AB, and Supplementary Figures S1, S2, S3, S4, S6, S7, S8, S9, S10, S11, S12. Please also ensure that figure legends in your manuscript include information on where the underlying data can be found, and ensure your supplemental data file/s has a legend.

Response: We have now included a "Source data file" ("S1_Data") spreadsheet to provide this information. This spreadsheet includes 7 "Source data" tabs, and the first tab of the spreadsheet indicates which source data tab corresponds to which figure (main or supplementary).
